# The Role of Liver Disease in Alcohol-Induced Cognitive Defects

**Published:** 1995

**Authors:** Roger F. Butterworth

**Affiliations:** Roger F. Butterworth, Ph.D., is director of the Neuroscience Research Unit at Hôpital Saint-Luc and professor of medicine and psychiatry at the University of Montreal, Montreal, Canada

**Keywords:** liver cirrhosis, liver function, cognitive process, brain, encephalopathy, organ transplantation, neuron, toxins, neurotransmitters

## Abstract

Chronic severe liver disease (i.e., cirrhosis) is a common complication of chronic alcohol abuse. Cirrhosis can cause brain dysfunction, most notably the disorder portal-systemic encephalopathy (PSE), which is characterized by cognitive and motor dysfunction and, eventually, by hepatic coma. Restoration of normal liver function—for example, through liver transplantation—can reverse some of the symptoms of PSE. PSE is caused by the shunting of venous blood into the general circulation without prior detoxification in the liver, allowing neurotoxins to reach the brain. The most prominent neurotoxin is ammonia. It interferes with the function of different brain cells and the communication between them. Ammonia and other toxins reaching the brain in PSE patients affect several neurotransmitters, including glutamate and monoamines.

Cognitive dysfunction is common among alcoholics; up to 75 percent of detoxified long-term alcohol-dependent patients show symptoms of cognitive impairment (Arria et al. 1990). Several factors have been proposed to contribute to alcohol-induced cognitive dysfunction. They include direct toxic effects of alcohol on nerve cells; compromised nutritional status (particularly thiamine [i.e., vitamin B] deficiency; see article by Langlais, pp. 113–121); and alcohol-induced damage to other organs, such as the liver.

Chronic alcohol abuse is the most important cause of liver disease in the United States. For example, about two-thirds of patients suffering from medical consequences of chronic liver disease have co-occurring diagnoses of alcohol abuse or dependence (Dufour and Caces 1993). A growing body of evidence suggests that alcohol-induced liver disease also plays an important role in precipitating the cognitive impairment encountered in alcohol-dependent patients and compounds the alcohol’s neurotoxic effects. For example, alcoholics with cirrhosis exhibit greater impairment in writing speed and reaction times than do alcoholics without liver disease (Gilberstadt et al. 1980). Similarly, when brain functions of alcoholics with and without cirrhosis were studied by such methods as measuring the blood flow through the brain or performing an electroencephalogram (EEG), patients with cirrhosis showed greater impairment than did patients without liver disease (Tarter et al. 1993). Finally, among alcoholic patients with various stages of cirrhosis, their performance on neuropsychological tests correlated with the severity of their liver disease (Pomier Layrargues et al. 1991).

This article focuses on the symptoms and causes of the most prevalent liver disease-associated brain dysfunction, portal-systemic encephalopathy (PSE). Although PSE also can be the consequence of nonalcoholic liver disease (i.e., cirrhosis), at least 50 percent of PSE cases result from alcoholic cirrhosis. Therefore, unless specifically mentioned otherwise, terms such as “liver damage” or “cirrhosis” refer to both the alcoholic and nonalcoholic disorder. In alcohol-dependent PSE patients, however, it is assumed PSE results from alcohol-induced liver damage.

This article describes how liver dysfunction may lead to PSE by causing alterations in several pathways of communication between nerve cells. The article also addresses the effectiveness of one potential treatment—liver transplantation—for liver disease and the PSE resulting from it.

## Portal-Systemic Encephalopathy

PSE, which is a common complication of cirrhosis (Butterworth 1994*a*), develops slowly and progresses through several distinct clinical stages that are characterized by specific cognitive and neuromuscular symptoms (table 1). The earliest symptoms include alterations of sleep patterns, reduced attention span, anxiety, depression, and muscle incoordination. As the condition progresses, additional symptoms appear, such as personality changes, memory loss, asterixis,^1^ confusion, stupor, and muscle rigidity. The disorder’s final stage is characterized by hepatic coma. The progression of PSE, which can happen rapidly, can be brought on by various factors, such as deterioration of the patient’s general health, gastrointestinal bleeding, infections, kidney failure, or use of sedative drugs (Arria et al. 1990).

As a result of the wide spectrum of psychiatric and neuromuscular symptoms encountered in alcohol-dependent patients with PSE, physicians often fail to diagnose the disorder correctly or even to recognize alcohol’s contribution to the development of the disorder. In the past, misdiagnoses have included depression, schizophrenia, mild forms of mania, and Parkinson’s disease (Tarter et al. 1986). The PSE diagnosis is complicated further by the fact that not all patients with PSE have obvious signs of liver disease and abnormal liver test results. Therefore, a physician may attribute brain dysfunction symptoms in alcohol-dependent patients to alcohol’s direct neurotoxic effects on the brain rather than to the involvement of alcoholic liver disease.

### Subclinical PSE

Prior to the development of overt PSE, many patients with less severe liver damage suffer from latent or subclinical PSE. These patients show no obvious clinical signs of brain dysfunction during routine neurological evaluation. Sensitive neuropsychological tests, however, can detect subtle signs of cognitive impairment (e.g., impaired memory capacity or psychomotor performance). Therefore, neuropsychological tests should be performed routinely on patients with severe liver damage, especially because abnormal test results may indicate a risk for impaired performance—for example, while driving an automobile (see below).

The fact that the liver disease, whether alcohol induced or nonalcoholic, contributes to the development of subclinical PSE has been demonstrated in several studies, as described below:

- Schomerus and colleagues (1981) compared the performance of 40 patients with either alcohol-induced or nonalcoholic cirrhosis with a control group of patients with alcoholic pancreatitis in a battery of tests of cognitive and motor function used to assess the ability to drive an automobile. Whereas 80 percent of patients with alcohol-induced cirrhosis were considered unfit to drive, only 25 percent of the patients with alcoholic pancreatitis had the same level of impairment. Patients with alcoholic cirrhosis consistently were more impaired than were patients with nonalcoholic cirrhosis; however, the difference in impairment was due to a greater degree of liver dysfunction rather than to the duration and quantity of previous alcohol consumption among the alcoholic patients.
- Tarter and colleagues (1984*a*) found that patients with nonalcoholic cirrhosis showed no overt symptoms of PSE but exhibited impaired visual scanning abilities, visuospatial capacity, and perceptual motor speed when more sensitive tests of cognitive functioning were used.
- A study of 22 patients with alcohol-induced cirrhosis, 20 patients with nonalcoholic cirrhosis, and 42 healthy control subjects matched for age and educational background assessed the subjects’ performance on several neuropsychological tests (Pomier Layrargues et al. 1991). All patients with cirrhosis had similar levels of liver dysfunction and none showed signs of overt PSE. Among the patients with cirrhosis, 76 percent failed at least two of the tests, whereas none of the controls failed any of the tests. No significant difference existed in the degree of cognitive impairment between alcoholic and nonalcoholic cirrhotic patients (table 2).

## Causes of PSE

PSE is caused by the shunting of venous blood, which contains metabolic products from the intestine, into the general circulation without passing through the liver first. The liver normally removes toxic substances and metabolic products from the blood (for more information on normal liver function, see sidebar p. 127). Consequently, if venous blood is shunted around the liver, toxic substances can reach other parts of the body, including the brain. One common neurotoxin is ammonia, which primarily is produced as a byproduct of protein digestion. Another potential toxin usually removed by the liver is the metal manganese (Pomier Layrargues et al. 1995). The accumulation of ammonia and other substances, such as manganese, in the brain may interfere with the actions of different neurotransmitters that mediate normal communication between nerve cells. Through this interference, these substances, and especially ammonia, contribute to the cognitive and neuromotor symptoms associated with PSE.

**Liver Function and Alcohol-Induced Liver Disease**

The liver is the largest internal organ of the human body. It performs several vital functions in the digestion, processing, and storage of nutrients and in the excretion of waste. One important liver function is the removal of harmful or toxic substances from the body. These include toxins that may be contained in some foods; toxic degradation products of nutrients; and other toxins, such as pesticides and alcohol and other drugs.

In many cases, liver enzymes will break down or transform toxic substances into less harmful products, which then can be either returned into the circulation or excreted in the urine. Other toxins, however, cannot be degraded or detoxified because the liver is lacking the appropriate enzymes. These toxins (e.g., the pesticide DDT) are then stored in the liver.

One of the toxins normally eliminated by the liver is ammonia. It is produced in the body primarily as a degradation product in the metabolism of dietary proteins and their building blocks, the amino acids. Ammonia is transported in the unoxygenated venous blood from the intestinal region to the liver through the portal vein. In the liver, the ammonia is transformed into urea, which is less toxic than ammonia and can be excreted safely with the urine.

**Alcohol and Liver Disease**

The liver also is the primary site for alcohol metabolism. Consequently, the liver is highly susceptible to alcohol-induced injury. The susceptibility of a person to alcoholic liver disease is determined by several factors (National Institute on Alcohol Abuse and Alcoholism [NIAAA] 1994). For example, a person’s gender, race, and genetic makeup seem to be contributing factors. Similarly, the duration and quantity of alcohol consumption contribute to the risk for liver disease.

During alcohol metabolism, some potentially toxic degradation products—such as acetaldehyde or highly reactive oxygen molecules (radicals)—are generated. In addition to alcohol itself, these products contribute to alcohol-induced liver damage.

Alcoholic liver disease usually progresses in three distinct stages, described below:

- Fatty liver or alcoholic steatosis: This initial stage of liver injury is found in about 90 percent of heavy drinkers (NIAAA 1994). Fatty liver is a reversible condition that is accompanied by only slight tissue damage. At this stage, little evidence of liver dysfunction is detected.
- Alcoholic hepatitis and fibrosis: These two disorders, which can be found in about 40 percent of heavy drinkers (NIAAA 1994), represent more serious conditions than fatty liver. They are associated with increasing destruction of liver tissue and, consequently, with increasing liver dysfunction. Alcoholic hepatitis results in inflammation and destruction of the liver tissue. In alcoholic fibrosis, healthy liver tissue is replaced by scar tissue.
- Alcoholic cirrhosis: This final stage of alcohol-induced liver disease is diagnosed in 15 to 30 percent of heavy drinkers (NIAAA 1994). A cirrhotic liver is characterized by heavily scarred liver tissue and a lumpy (nodular) appearance. Coinciding with the structural damage is severe dysfunction, which in turn can impair the functioning of other organs and contribute to disorders such as kidney failure; gastrointestinal bleeding; and brain disorders, including portal-systemic encephalopathy.

The treatment of alcoholic liver disease first and foremost requires abstinence from alcohol to prevent further damage. Because the liver has the capacity to regenerate itself (Diehl 1993), some patients may recover from alcohol-induced liver damage—especially in the early stages—without further treatment. For patients with end-stage cirrhosis, however, liver transplantation may be the only treatment option (NIAAA 1994).

—*Susanne Hiller-Sturmhöfel*

*Susanne Hiller-Sturmhöfel, Ph.D., is a science editor of* Alcohol Health & Research World.

ReferencesDiehlAMEffects of alcohol on liver regenerationAlcohol Health & Research World1742792831993National Institute on Alcohol Abuse and AlcoholismEighth Special Report to the U.S. Congress on Alcohol and HealthNIH Pub. No. 94–3699Bethesda, MDNational Institutes of Health1994

**Figure 1:**
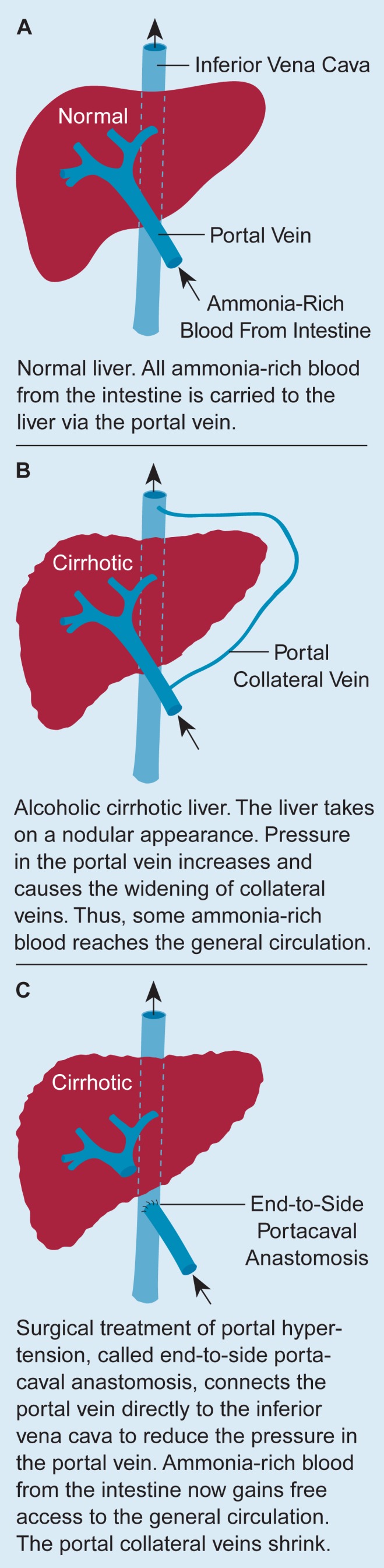
Distribution of ammonia-rich blood in normal and cirrhotic liver.

Under normal circumstances, venous blood is transported to the liver through the portal vein (figure 1A). Before reaching the liver, the portal vein crosses another major vein, the inferior vena cava, which transports unoxygenated blood from the lower limbs and the abdomen to the heart. In patients with alcohol-induced or nonalcoholic cirrhosis, pressure in the portal vein is increased due to obstructions in the liver. This phenomenon is referred to as portal hypertension. Portal hypertension can result in life-threatening complications, such as gastrointestinal bleeding.

Portal hypertension can lead to the shunting of blood from the portal vein into the inferior vena cava through two mechanisms (figure 1B and 1C). First, portal hypertension can cause the dilation (i.e., widening) of minuscule blood vessels, so-called collateral veins, that connect the portal vein and the inferior vena cava. This allows some ammonia-rich blood to enter the general circulation without passing through the liver and to reach the brain.

Additionally, in patients with severe portal hypertension, shunts between the portal vein and the inferior vena cava may have to be created through surgery to relieve the hypertension. One such procedure—called portacaval anastomosis—that creates a shunt outside the liver is depicted in figure 1C. Another more recent technique that is gaining popularity is the transjugular intrahepatic portosystemic stent shunt (TIPS), which creates a shunt inside the liver. Both surgical techniques cause ammonia-rich blood from the portal vein to enter the general circulation. This leads to PSE in up to 30 percent of patients treated with TIPS. For example, in one study of 108 patients with alcohol-induced and nonalcoholic cirrhosis, TIPS treatment precipitated or worsened PSE in 24 percent of the patients. Eleven patients required hospitalization because of severe PSE; 3 patients even fell into hepatic coma and died (Skeens et al. 1995). Thus, PSE can be the consequence of liver disease per se or can be caused or exacerbated by a life-saving surgical procedure designed to prevent the immediate danger of potentially fatal hemorrhage.

### The Role of Ammonia in PSE

**Figure 2:**
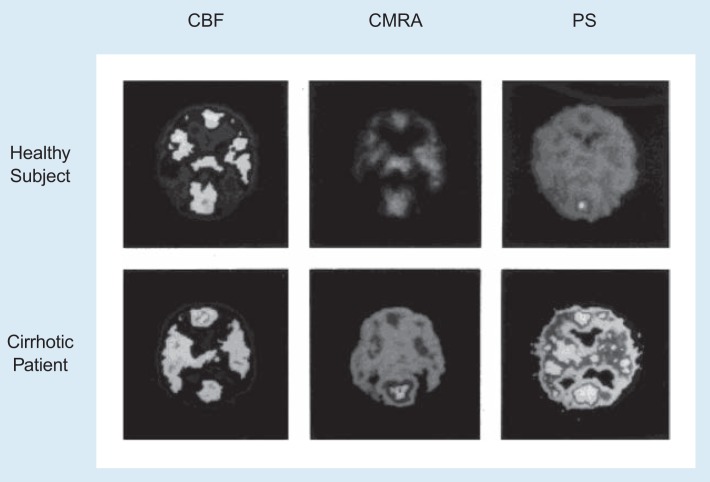
Use of positron emission tomography (PET) in the evaluation of portal-systemic encephalopathy (PSE) in alcoholic liver disease. Shown are PET images of the cerebral blood flow (CBF; a measure of brain function), the cerebral metabolic rate for ammonia (CMRA; the rate with which the brain takes up and removes ammonia), and the permeability/surface area product (PS; a parameter that reflects how easily ammonia can enter the brain) from a healthy subject and a cirrhotic patient with mild PSE. (From Lockwood et al. 1991, with permission.)

As mentioned earlier, the concentration in the brain of the neurotoxin ammonia rises as a consequence of severe liver dysfunction. The contribution of excess ammonia to the development of PSE recently has been demonstrated by an imaging technique called positron emission tomography (PET). This technique allows scientists to monitor the metabolism of different substances in the brain. PET studies in alcoholics with cirrhosis and early stages of PSE revealed that compared with healthy subjects, the brain in alcoholics showed increased uptake and removal of ammonia (figure 2; Lockwood et al. 1991).

Ammonia exerts its toxic effects on the brain through several mechanisms. For example, ammonia concentrations similar to those achieved in laboratory animals with experimentally induced liver failure directly interfere with both inhibitory and excitatory neurotransmitter systems in the brain (Raabe 1989; Fan et al. 1990). (For a more detailed discussion of different transmitter systems, see sidebar, p. 128.)

**Signal Transmission Among Nerve Cells**

Communication or signal transmission among nerve cells (neurons) or between neurons and the cells of other organs underlies such diverse functions as movement or thought. This communication is achieved through a combination of electric and chemical processes. Within a neuron, an electric signal travels along the length of the cell. Between cells, molecules called neurotransmitters primarily mediate signal transmission.

**Signal Transmission Across Synapses**

Neurons generally are separated from each other by microscopic gaps called synapses. Accordingly, a cell emitting a signal is referred to as a presynaptic cell; a cell receiving a signal is called a postsynaptic cell. Although synapses are very small, they cannot be crossed by an electric signal, and neurotransmitters are used to convey the signals. Neurotransmitters are stored in small vesicles in the presynaptic nerve cell ending. Although there are different neurotransmitters in the body, each neuron releases predominantly one kind of neurotransmitter.

When an electric signal arrives at the end of the presynaptic cell, the storage vesicles discharge their neurotransmitter contents into the synapse. The molecules diffuse across the synapse to the postsynaptic cell. This cell’s membrane contains protein molecules called receptors, to which the neurotransmitters can bind. Each receptor is shaped so that it only binds one specific neurotransmitter; however, several different receptor molecules may exist for each neurotransmitter. Also, each cell may contain receptors for several neurotransmitters.

Neurotransmitters bind to their receptors without actually entering the postsynaptic cell. The binding reaction triggers a chain reaction of events in the postsynaptic cells. The reaction is terminated when the neurotransmitter dissociates from its receptor to be degraded or to be taken back up into storage vesicles in the presynaptic cell.

**Neurotransmitters**

As mentioned earlier, several different neurotransmitters exist. They fall into categories according to their effects on the postsynaptic cells or according to their chemical structure.

Neurotransmitters can have an excitatory or inhibitory effect on the postsynaptic cell. Excitatory neurotransmitters may stimulate the cell to produce an electric signal of its own, thus transmitting the incoming signal further along the cell and to adjacent cells. Inhibitory neurotransmitters, in contrast, reduce the postsynaptic cell’s sensitivity to other incoming signals, thereby preventing further signal transmission. Each postsynaptic cell receives signals from many other neurons. Some signals are transmitted by excitatory neurotransmitters and others by inhibitory ones. The ultimate response of the post-synaptic cell depends on the combined effect of all signals.

Several substances can function as neurotransmitters. Many of them are small molecules that rapidly transmit signals among nerve cells and to other cells, such as muscle cells. These neurotransmitters can be subgrouped according to their chemical structure into several categories, three of which (described below) are most prevalent in the body and may mediate alcohol’s effects.

- Acetylcholine, one of the most common neurotransmitters, is in a group by itself. It is secreted by many neurons in the brain and also by neurons innervating the muscles. In most cases, acetylcholine has an excitatory effect on the postsynaptic cell.
- Monoamines are compounds that contain a characteristic chemical group. Several of these substances function as neurotransmitters, including dopamine and serotonin. Dopamine usually has an inhibitory effect on the postsynaptic cells. Neurons using dopamine as a transmitter are involved in controlling motor activity and the rewarding effects of abused drugs, such as alcohol. Serotonin-mediated transmission affects mood, sleep, the consumption of drugs, the development of tolerance to alcohol and other drugs, and the sensation of pain.
- Some amino acids not only are building blocks of proteins but also function as neurotransmitters. One example is glutamate, which serves as an excitatory neurotransmitter for sensory signals.

The accurate functioning of all neurotransmitter systems is essential to ensure the normal brain activities required for every aspect of human body function. Alcohol interferes with the intricate network of neural signal transmission through numerous pathways, thus causing the cognitive and motor impairments that are observed in many alcohol-dependent people (National Institute on Alcohol Abuse and Alcoholism 1994).

—*Susanne Hiller-Sturmhöfel*

*Susanne Hiller-Sturmhöfel, Ph.D., is a science editor of* Alcohol Health & Research World.

ReferencesNational Institute on Alcohol Abuse and AlcoholismEighth Special Report to the U.S. Congress on Alcohol and HealthNIH Pub. No. 94–3699Bethesda, MDNational Institutes of Health1994

Chronic exposure of the brain to ammonia also causes structural and functional changes in specific brain cells called astrocytes. These cells, among their other functions, are involved in the metabolism of neurotransmitters and other substances required for nerve cell function. For example, astrocytes are essential for removing excess amounts of the excitatory neurotransmitter glutamate. Impairment of glutamate-mediated neurotransmission may contribute to the cognitive dysfunction characteristic of PSE (Butterworth 1993).

In addition, evidence suggests that chronic liver disease and the resulting accumulation of neurotoxins, such as ammonia, in the brain contribute to PSE development by interfering with the actions of other neurotransmitters. For example, early PSE symptoms include altered sleep patterns, personality changes, depression, and paranoia. These symptoms have been attributed in part to the dysfunction of neurotransmitters called monoamines, such as serotonin or dopamine. Consistent with these observations, researchers have detected changes in the metabolism of monoamine neurotransmitters in patients with PSE.

### Serotonin Deficiency

Several studies indicate that PSE patients have a serotonin deficit in the brain, which may contribute to the PSE symptoms. For example, PSE patients with alcohol-induced cirrhosis who died in hepatic coma showed a significant increase in the numbers of receptors for serotonin on nerve cells in some areas of their brains (Raghavendra Rao and Butterworth 1994). Such an increase in receptor numbers often occurs when the concentration of the ligand (i.e., the substance binding to the receptor) is too low. The increase may reflect the organism’s effort to saturate the cells with binding sites so that every last ligand molecule can be bound. Consequently, an increase in serotonin receptors may be indicative of a serotonin deficiency in the brains of PSE patients.

Clinical data also support a serotonin deficit in PSE patients: When patients with cirrhosis, but without PSE, received the serotonin antagonist ketanserin to lower their portal hypertension,^2^ a significant number of them developed PSE (Vorobioff et al. 1989). Ketanserin inhibits the binding of serotonin to its receptor and thus mimicks a serotonin deficiency. If this artificial serotonin deficiency can lead to PSE, then a cirrhosis-induced serotonin deficiency also may contribute to PSE.

The mechanisms underlying changes in serotonin levels have not been resolved conclusively. Ammonia levels are one factor affecting serotonin levels: In order to remove toxic ammonia from the brain, it is incorporated into the amino acid glutamine (Jonung et al. 1983). Elevated ammonia concentrations therefore result in higher glutamine concentrations. Glutamine, in turn, facilitates the uptake of the amino acid tryptophan, which is a precursor molecule of serotonin (Jonung et al. 1983). When analyzing the serotonin metabolism of patients in hepatic coma, researchers found that tryptophan levels are higher than normal in both the brain and the cerebrospinal fluid (CSF) (Young et al. 1975).

Conversely, a study by Bergeron and colleagues (1990) detected increased concentrations of a serotonin degradation product in both the brain and CSF of PSE patients with cirrhosis and in the brains of laboratory animals with experimentally induced PSE. This increase already is apparent early in the progression of neurological symptoms of PSE. The increase in the levels of both serotonin precursors and serotonin degradation products indicates that in the brains of PSE patients, serotonin may be produced at a higher rate but is degraded even faster. Combined, these two processes could result in lower overall levels of serotonin in the patients’ brains.

### Impaired Dopamine Function

**Figure 3:**
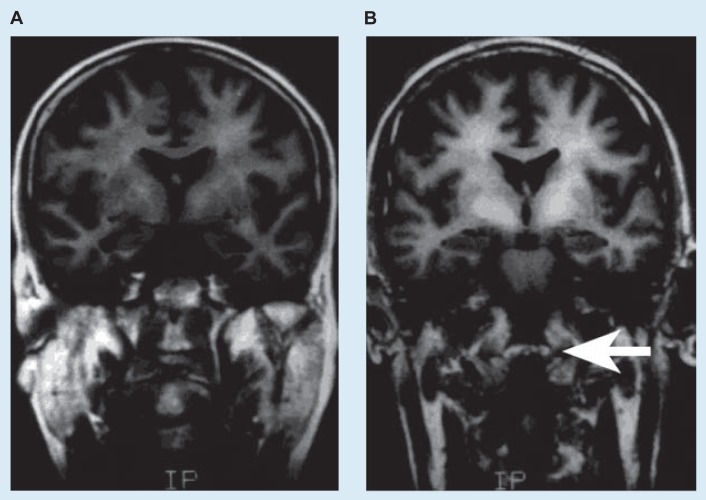
Magnetic resonance imaging of the pallidum in a healthy subject and in a patient with cirrhosis and portal-systemic encephalopathy. In the healthy subject (A), the pallidum has a normal appearance; in the patient (B), manganese accumulation produces an intense signal in the pallidum (indicated by arrow). (From Kulisevsky et al. 1992, with permission.)

The normal function of the neurotransmitter dopamine also appears to be affected in PSE patients (Bergeron et al. 1989). Alterations of dopamine-mediated neurotransmission are the most likely causes of the motor impairment (e.g., tremor and rigidity) that sometimes is observed in PSE patients. Researchers have discovered a significant loss of dopamine receptors on the postsynaptic cells in the pallidum (a brain structure involved in motor control) of PSE patients (Mousseau et al. 1993). Also, using magnetic resonance imaging (MRI), Kulisevsky and colleagues (1992) found an enhanced signal in the pallidum of PSE patients (figure 3). One possible cause for the MRI signal is the accumulation of manganese in the brains of these patients (Pomier Layrargues et al. 1995). Manganese is known to be toxic to dopaminergic neurons (Bird et al. 1984).

## Neuropsychiatric Status After Liver Transplantation

Liver cirrhosis is a permanent disorder that cannot be reversed except by liver transplantation. Several studies have investigated whether liver transplantation can reverse PSE symptoms. In an early study, Parkes and colleagues (1970) compared the neuropsychiatric status and brain activity before and after transplantation in four patients with serious liver disorders, one of them with chronic PSE. All patients showed striking and lasting clinical improvement of their cognitive impairments as well as normalization of their EEG patterns following liver transplantation.

Tarter and colleagues (1984*b*) evaluated the cognitive and psychiatric status of 10 patients with nonalcoholic cirrhosis 3 years after successful liver transplantation. In all patients, measures of intelligence, verbal skills, attention, spatial organization, memory, and learning had improved after transplantation. In a similar study, Arria and colleagues (1991) compared the cognitive functioning of patients with alcoholic cirrhosis shortly before and 1 year after liver transplantation. Most of the patients showed significant improvement in their psychomotor function, visuospatial capacity, attention, and perception skills. Tests assessing their memory capacity, however, showed no significant improvement, indicating that non-liver disease-related factors may be responsible for memory impairment.

These results confirm that alcohol-induced liver disease contributes to cognitive dysfunction in alcoholics and that in many patients, this dysfunction is at least partially reversible—for example, by liver transplantation. Some aspects of cognitive impairment may not be reversible by the normalization of liver functions if factors other than liver disease, such as alcohol’s neurotoxicity or alcohol-induced thiamine deficiency (see article by Langlais, pp. 113–121), have contributed to the impairment of these cognitive functions.

## Conclusions

PSE is a complication of both alcoholic and nonalcoholic cirrhosis that results in cognitive impairment (including attention deficits, personality changes, memory loss, and confusion) and neuromuscular impairment (including incoordination and tremors). In alcoholics, cirrhosis and the PSE resulting from it compound the toxic effects that alcohol exerts directly on the brain.

Because cirrhosis is a chronic disorder, alcoholic cirrhosis may perpetuate and magnify alcohol-induced cognitive impairment even after the patient stops drinking (Tarter et al. 1993). However, reversal of the liver dysfunction—for example, through liver transplantation—can result in the normalization of many cognitive deficits. In addition, treatment with ammonia-lowering drugs, such as lactulose and the antibiotic neomycin, usually improves PSE symptoms in patients with less severe liver dysfunction.

During the last decade, much interest has focused on the possibility that endogenous compounds with benzodiazepine (valium)-like properties, which are present in the blood and brains of a subgroup of PSE patients, may contribute to PSE (Olasmaa et al. 1990). This hypothesis has led to clinical trials in which PSE patients were treated with flumazenil, a drug with potent antibenzodiazepine effects. Up to 30 percent of the patients responded positively to flumazenil administration (Pomier Layrargues et al. 1994).

PSE is characterized by many diverse symptoms, which also resemble the consequences of other disorders; as a result, the disorder often is not diagnosed correctly. Therefore, physicians should carefully assess liver function in all alcoholics with neuropsychological impairment.

Although it seems clear that the failure of the liver to remove toxic ammonia from the blood is an important factor in precipitating PSE, the exact mechanisms of how ammonia affects brain functions still require further investigation. Furthermore, the contribution of alterations in the functions of different neurotransmitters (i.e., monoamines and others) to the symptoms and consequences of PSE are not known in detail. A better understanding of the molecular processes involved may open new avenues to treating cognitive impairment in alcoholic patients.

Similarly, it is important to study other aspects of alcoholic liver disease that may contribute to PSE. For example, it has been suggested that liver dysfunction may lead to nutritional vitamin deficiencies, including vitamin E and some B vitamins (Butterworth 1994*b*). Alcohol-related deficiencies in these vitamins have been implicated in several neuropsychological and neuromotor dysfunctions in alcoholics (Arria et al. 1990, 1991). Together, these findings underscore the importance of looking beyond the neurotoxic actions of alcohol per se when trying to identify the causes of cognitive impairment in alcoholic patients.

## Figures and Tables

**Table 1:** The Different Stages of Portal-Systemic Encephalopathy (PSE) and Their Characteristic Cognitive and Neuromuscular Symptoms

Stage of PSE	Cognitive Symptoms	Neuromuscular Symptoms
Subclinical	Abnormal psychometric test scores	—
Grade 1	Abnormal sleep patterns, shortened attention span, irritability, apathy	Tremor, incoordination
Grade 2	Personality changes, disorientation (time), memory loss	Asterixis,^1^ dysarthria,^2^ abnormal muscle tone
Grade 3	Confusion, drowsiness, sleepiness, paranoia, anger, stupor	Hyperactive reflexes, muscle rigidity
Grade 4	Coma	—

1 Asterixis is a neurological disorder characterized by involuntary jerking movements, especially of the hands.

2 Dysarthria is an impairment of the ability to articulate due to emotional stress, brain injury, or paralysis or incoordination of the muscles used for speaking.

**Table 2:** Demonstration of Subclinical Hepatic Encephalopathy in Alcoholic and Nonalcoholic Cirrhotics by Neuropsychological Testing

Test	Score		

	Controls	Cirrhotics	
	
	*n* = 42	Alcoholic *n* = 22	Nonalcoholic *n* = 20
Delayed recall^1^ (number of words)	2.7 ± 0.5	2.2 ± 0.9*	2.3 ± 0.9*
Serial 3’s^2^ (time in seconds)	57.0 ± 28.0	81.0 ± 45.0*	78.0 ± 39.0*
Arithmetic^3^ (number of errors)	0.6 ± 0.9	2.5 ± 1.9*	2.2 ± 1.9*
Recall months backwards^4^ (time in seconds)	14.0 ± 6.0	33.0 ± 34.0*	28.0 ± 30.0*
Digit span forward^5^ (number)	6.5 ± 1.2	5.7 ± 1.1*	5.9 ± 1.2*
Digit span backward^5^ (number)	4.7 ± 0.9	3.9 ± 1.0*	4.2 ± 1.4*
Alphabet 6 (time in seconds)	9.4 ± 4.2	17.0 ± 13.0*	17.0 ± 11.0*
Digit symbol^7^ (number)	39.6 ±11.4	25.7 ± 8.3*	27.7 ± 10.2*
Reitan trail-making test 8			
part A (time in seconds)	38.9 ± 13.5	61.6 ± 24.3*	59.7 ± 22.1*
part B (time in seconds)	83.9 ± 31.8	140.1 ± 50.1*	153.5 ± 50.2*

1 Subjects have to recall words given to them 10 minutes earlier.

2 Subjects count forward by 3’s, from 1 to 100.

3 Subjects mentally perform eight calculations of increasing difficulty.

4 Subjects list the months of the year in reverse order.

5 Subjects have to recall immediately digits from a previously given list.

6 Subjects recite, in order, the letters of the alphabet.

7 Subjects must associate a set of symbols with a set of numbers.

8 Subjects connect, in order, a series of numbers (part A) or numbers and letters (part B) randomly presented.

NOTE: Values indicated are the mean ± standard error; * indicates significant differences from control values (*p* < 0.01). The differences between scores for alcoholic and nonalcoholic cirrhotics were not statistically significant (adapted from Pomier Layrargues et al. 1991).

## Footnotes

1 For definitions of this and other clinical and technical terms, see central glossary on pp. 136–137.

2 Among other functions, serotonin has been implicated in blood pressure control.
